# Expanding Representation of Low and Middle Income Countries in Global Dementia Research: Commentary From the Alzheimer's Association

**DOI:** 10.3389/fneur.2021.633777

**Published:** 2021-03-15

**Authors:** Claire Sexton, Heather M. Snyder, Lakshmi Chandrasekaran, Susan Worley, Maria C. Carrillo

**Affiliations:** ^1^Alzheimer's Association, Chicago, IL, United States; ^2^Independent Science Writer, Bryn Mawr, PA, United States

**Keywords:** Alzheimer's, dementia, convening, funding, collaboration, public health

## Abstract

Alzheimer's disease (AD) and all other dementia represent a global challenge, with an estimated 50 million individuals in the world living with dementia today. In low and middle income countries (LMICs), the burden of disease often is greater, and some of these countries are projected to have some of the largest increases in dementia prevalence during the next few decades. As the world's largest voluntary health organization dedicated to AD and all other dementia, the Alzheimer's Association is committed to its vision of a world without dementia and recognizes the needs, challenges, and opportunities for dementia research in all parts of the world, and especially in LMICs. Currently, the Association is devoting more than $215 million in funding to nearly 600 best-of-field projects in 31 countries, including a significant number of projects that advance and support LMIC-specific research. The innovative work in LMICs is focused on addressing unmet needs or challenges associated with the many unique cultural, demographic, and economic characteristics of these countries. The Association also is expanding leading global forums such as the Alzheimer's Association International Conference (AAIC). In an effort to create new learning and participation opportunities, the Association also has been partnering with other international organizations and collaborating with local leadership to provide AAIC Satellite Symposia (AAIC SS) in LMIC regions around the world. In 2021 and beyond, the Association is committed to continuing these LMIC-focused initiatives, identifying gaps in LMIC research and resources, and enhancing collaboration and communication among researchers in these regions.

## Introduction

The need for global coordinated research that will reach and benefit all communities around the world has perhaps never been greater. For several decades, in response to the global challenge of dementia, the Alzheimer's Association has recognized that all countries are critical and integral components of international research efforts in the global mission to eradicate dementia. Accordingly, the Association is dedicated to ensuring complete representation of those countries with a World Bank designation of middle or lower middle income (1), many of which have a projected prevalence of dementia that is much higher than the anticipated prevalence in higher income countries (2, 3).

Since its founding in 1980, the Alzheimer's Association has had an increasingly global focus. This focus has included a growing awareness of the needs of low-income and middle-income countries (LMICs), as well as the importance of their inclusion in the global research enterprise—an awareness initially informed in large part by the work of the 10/66 Dementia Research Group (DRG). This research group, established in 1998 as a part of Alzheimer's Disease International (ADI), brought together researchers with a special focus on LMICs and succeeded in challenging the belief, prevalent in the 1990s, that dementia was relatively rare in these countries (4, 5). The name of the research group reflected an imbalance at that time such that ~66% of all people with dementia lived in LMICs, yet only about 10% of population-based research was being conducted in these regions (4). Due largely to the efforts of the 10/66 DRG, ADI noted that within a decade this disparity had lessened, so that by 2009 ~39% of dementia prevalence studies were conducted in LMICs, including 18 studies in Latin America and the Caribbean (6).

In subsequent years, the Alzheimer's Association has supported and joined in efforts by other international organizations, including ADI and the World Health Organization, to significantly advance our understanding of and approach to LMICs and to help inform strategies for addressing dementia by countries around the world (7–9). By 2013, a steadily growing awareness of the need for full global cooperation in the effort to combat dementia led to a gathering of the G8 countries in London, where leaders developed a multinational response to the crisis (10). During the same period, the Alzheimer's Association and other leading global organizations began to strongly encourage the development of national or country-wide plans for addressing dementia (7, 8). By 2015, the WHO published an international “Call for Action” recommending that all countries develop national public health strategies aimed at reducing the impact of dementia (11), and in 2017 developed specific guidelines for approaching dementia intended for healthcare providers, governments, policy-makers, and other stakeholders, including those in LMICs (12).

In the absence of effective treatments for dementia, a guiding principle for all of these efforts has been to accelerate research with the aim of stopping or slowing symptomatic illness for as long as possible, while providing high-quality dementia care and supporting the well-being of individuals with symptoms and/or their carers. Efforts to prevent or post-pone dementia are likely to have the greatest benefit in low-income and middle-income countries, where most dementia occurs, and where individuals are most likely to encounter poverty, inequality, and limited access to health care (3). Yet the prevalence of AD and other dementia continues to increase at a rapid pace in many LMICs (2, 3). In response, the Alzheimer's Association, in collaboration with a wide range of international organizations, is redoubling its efforts to advance research around the globe. In a collaboration between the Alzheimer's Association and the National Institute on Aging (NIA), for example, the International Alzheimer's and Related Dementias Research Portfolio (IADRP) was established to both collate and categorize the dementia research portfolios of major funding organizations but also is focused on gaining a comprehensive assessment of the current landscape of AD research in all parts of the world (https://iadrp.nia.nih.gov/) (13). Through such partnerships, the Association aims to facilitate the global conversation about all aspects of dementia and ensure LMICs are well-represented in that conversation, with the goal of eventually reducing future prevalence of dementia, and in turn reducing its socioeconomic consequences.

This article contains a summary of existing Alzheimer's Association initiatives that support research in LMICs, spanning funding and partnering on research studies, as well as convening researchers both globally and regionally. While it is important to note that LMICs are not homogenous, and that funding and convening activities are unique in each country, we also provide examples in each section for illustrative purposes.

## Funding Critical Research/Pilot Studies

Since 1982, the Alzheimer's Association has supported a wide range of projects involving basic science, social and behavioral research and clinical research through numerous investigator-initiated peer-reviewed grant programs. These programs became international in 2000, at that time reaching researchers in more than 50 countries through their applications, awarded funding and review process (13). Today the Association has nearly 600 best-of-field projects, more than $215 M projects in 31 countries, including many dedicated to research in LMICs—with the examples that follow in this section all part of the Alzheimer's Association's portfolio of funded research studies.

A significant number of these projects, some of which are funded in partnership with the Global Brain Health Institute (GBHI) and Alzheimer's Society (UK), are conducted in LMICs by researchers who reside in these regions (14). Though these projects have a vast range in terms of scope and subject, they generally have the common goal of addressing challenges in these countries and regions of the world. Findings from these studies often are used to inform local and national health care policies. These projects—which have ranged from tailoring mental ability tests in the Democratic Republic of the Congo, to evaluating brain health among Syrian refugees after forced migration, to using digital media and film arts to augment dementia care and improve outcomes in India—emphasize the importance of re-evaluating assumptions derived from research conducted primarily in high-income countries (HICs), to develop diagnostic tests and other tools or resources that are free of cultural, socioeconomic, educational, and other biases (14).

Despite the current shift toward biological definitions of AD and other dementia (15), for example, instruments currently available in LMICs continue to rely heavily on psychometric evaluations and clinical interviews. Because many of the cognitive and functional assessment tools used in these regions were originally developed and validated in HICs (5), one goal is to adapt these tools so that they can be used more effectively in LMICs. In one current project, involving a collaboration between the University of Botswana and the University of California, San Francisco (UCSF), Dr. Lingani Mbakile-Mahlanza, D.Psyc, and her team are examining whether currently available computerized and paper-based tests used to diagnose dementia can be culturally adapted and validated to improve the diagnosis of individuals in Botswana. This study also is examining whether some risk factors for dementia, often identified in HICs, are relevant in sub-Saharan Africa (14). Another funded study, taking place in Egypt under the leadership of Dr. Rufus Akinyemi, PhD, MSc, MWACP, FMC, aims to determine whether universal social network platforms, such as Facebook, may be used to develop unbiased tools for identifying individuals at risk for developing dementia and developing pathways to early intervention (14). In Belo Horizonte, Brazil, Dr. Elisa de Paula França Resende, MD, of Universidade Federal de Minas Gerais—Faculdade de Medicina, and her team are exploring the effects of basic literacy acquired late in life on potential improvements in brain connectivity and memory, a subject that has been understudied among elderly populations with low educational attainment in low-income countries (14). And in Turkey, Dr. Derya Durusu Emek Savas and their team are exploring unique cognitive training strategies that may help to improve cognition in individuals with mild cognitive impairment (MCI), and potentially delay progression from MCI to AD (14).

In the absence of curative interventions for dementia, caregiving and the well-being of caregivers is another important focus of dementia-related care and research supported by these investigator-initiated grants. In Guadalajara, Mexico, for example, Dr. Brenda Perez Cerpa, MD, is funding the evaluation of a decision aid for families of individuals with advanced dementia (14). The aim of the project is to determine how this tool might improve the quality of connections between families and healthcare providers, and in turn improve end-of-life care.

## Larger Collaborative Studies

The Alzheimer's Association is devoted not only to developing but also to expanding multinational studies and projects—from international clinical trials to prevention, biomarker validation, and health care utilization studies—to ensure that they include genetically, ethnically, and culturally diverse populations. For some well-established studies already underway, the Association is committed to providing additional funding to ensure greater inclusion of LMICs. One such multinational study is the Dominantly Inherited Alzheimer Network Trials Unit (DIAN-TU), based at Washington University School of Medicine in St. Louis (16). The DIAN-TU is the world's first prevention trial platform for at-risk families with dominantly inherited Alzheimer's disease (DIAD). This series of interventional therapeutic trials is evaluating the safety, tolerability, and effectiveness of drugs that have the potential to prevent, delay, or possibly reverse changes in the brain associated with dementia. DIAN-TU is led by director and principal investigator Randall J. Bateman, MD, the recipient of an Alzheimer's Association research grant for this project. Recently the Alzheimer's Association contributed funding to permit the inclusion of new sites throughout Central and South America, including in Argentina, Brazil, Colombia and Mexico. This expansion will be used to establish a multicenter registry of pre-symptomatic and symptomatic individuals in Latin America (both gene carriers and non-carriers), and will enable comparison of clinical, psychometric, neuroimaging and biomarker data from Latin American DIAD families with corresponding data from non-Latin American DIAD families (16).

A similarly large collaboration aimed at preventing dementia is the World Wide FINGERS network (17, 18), which was launched in 2017 and has brought together more than 30 participating countries in a global network of multimodal lifestyle intervention trials aimed at dementia risk reduction and prevention. These trials are modeled after the Finnish Geriatric Intervention Study to Prevent Cognitive Impairment and Disability (FINGER) trial, the first randomized controlled trial (RCT) to show that it is possible to prevent cognitive decline among older at-risk individuals using a multidomain lifestyle intervention (19). With support from the Alzheimer's Association, a Latin American first-of-its-kind study, LatAm-FINGERS, is in the final planning stages and will involve 1,300 subjects from Argentina, Bolivia, Brazil, Chile, Colombia, Costa Rica, Dominican Republic, Ecuador, Mexico, Paraguay, Peru, Puerto Rico, and Uruguay (17, 18). The study participants, individuals who are thought to be at a higher risk of cognitive decline due to a range of factors such as sedentary lifestyle, poor diet, and/or a suboptimal metabolic-cardiovascular profile, will be randomly assigned to one of two interventions. The study, which begins recruiting subjects in second quarter of 2021, will aim to evaluate both the feasibility and efficacy of a FINGER multi-domain lifestyle intervention across Latin America.

In January 2020, the Alzheimer's Association also joined leaders in dementia research from Latin America, the GBHI, the Tau Consortium, and the National Institutes of Health (NIH) in forming a new multinational consortium that likewise aims to expand dementia research in Latin America. Research Dementia, Latin America, or ReDLat, is designed to identify unique genetic, social, and economic determinants of health that contribute to the development of AD and other dementia in Latin America. By collecting and analyzing neuroimaging, genetic, and behavioral data from more than 4,000 individuals in Argentina, Brazil, Chile, Colombia, Mexico, Peru, and the US, ReDLat investigators expect to broaden our understanding of the genetic and environmental determinants of dementia, particularly in diverse and underserved populations in Latin America. This large-scale 5-year project is bringing together experts in the fields of neurology, neuropsychology, geriatrics, psychiatry, neuroscience, and genetics from across Latin America.

In July 2020, the Alzheimer's Association highlighted its work with researchers from all around the world, and led by the team at University of Texas, San Antonio, to support and advance critical research in response to the current pandemic crisis, with a special focus on research examining the neurological sequelae of SARS-CoV-2 following acute infection. Although much remains to be known about the long-term consequences of SARS-CoV-2 infection, several research studies suggest that COVID-19 is associated with neurological complications (20). Thus, there is an urgent need to investigate the downstream impact of COVID-19 on the brain, and address unanswered questions regarding the effects of SARS-CoV-2 on the cerebrovascular system. In this network, the Alzheimer's Association joins representatives from more than 30 countries, with technical guidance from the WHO, as a member of an international, multidisciplinary consortium dedicated to collecting and evaluating data on the short- and long-term consequences of the viral infection on the central nervous system (CNS), as well as examining differences across countries (21).

## International Meeting Forums and Organizations

The Alzheimer's Association has long been dedicated to fostering international collaboration among dementia researchers in all countries, in part by providing a range of inspiring forums and professional opportunities. The Alzheimer's Association International Conference (AAIC) is the world's largest gathering of researchers from around the world whose work is focused on AD and other dementia (22). As a part of the Alzheimer's Association's research program, AAIC serves as a catalyst for generating new findings about dementia and nurturing a vital, collegial research community. In July 2019, AAIC drew ~6,000 leading experts from 56 countries, including over 400 attendees from 23 LMICs, to Los Angeles to share leading basic science and clinical research discoveries, including new directions for the diagnosis, prevention, and treatment of dementia. In order to defray costs associated with AAIC registration, housing and travel expenses, 119 travel fellowships were awarded to researchers based in LMICs. These awards are competitive and based on financial need, with priority given to applicants who are scientists, early career investigators, post-doctoral fellows, or students based in LMICs.

In July 2020, in response to the global pandemic, AAIC was held virtually for the first time. This free, on-line conference attracted the meeting's largest audience to date, with more than 33,000 registered attendees and featuring more than 3,000 scientific presentations. The online accessibility of the AAIC experience further enabled dementia scientists from every corner of the world to share and discuss the latest research findings, and network to build new international collaborations, with over 7,000 registered attendees from 93 LMICs. A growing focus on LMICs was also evident in the many presentations that examined or highlighted issues faced by regions comprising these countries, including a plenary session led by Dr. Vijayalakshmi Ravindranath, a scientific leader from the Indian Institute of Science in Bengaluru, India. Such presentations included a systematic review of culturally tailored dementia interventions for minority ethnic groups in low- and middle-income countries, a description of a cognitive assessment test adapted and validated for use by older adults in a Brazilian indigenous community, an examination of multi-morbidity as a correlate of dementia among older people in Central Africa, an overview of socioeconomic determinants of dementia among Caribbean Hispanics, and an examination of cognitive dysfunction among middle-aged adults with type 2 diabetes in South Africa.

## Satellite Symposia

In December 2015, AAIC launched a series of satellite symposia to extend the reach of AAIC across the globe and provide more learning and participation opportunities to people from all countries, including LMICs. When the first satellite research symposium was held in Mexico City, the prevalence of dementia in Mexico, Brazil, the Andean Area, and Central America was expected to increase by more than 400% between 2010 and 2050 (6). Moreover, the Pan American Health Organization warned that problems associated with this increase would likely be compounded by a fragile health care infrastructure and lack of access to health services and sanitation in these regions. Yet the meeting convened at an auspicious time and on an optimistic note, as the first national dementia plan in a Spanish-speaking country had just been established in Mexico the previous year (6).

This first AAIC satellite symposium (AAIC SS) underscored the value of ensuring the incorporation of previously neglected LMIC cohorts in international studies, in part by highlighting unique characteristics of studies across Latin American populations, including those assessing prevalence and incidence of both risk factors and dementia (23). The results of epidemiologic studies and national surveys presented at the meeting (e.g., SABE) revealed unique challenges facing older Mexicans and other Latinos with dementia, such as low literacy and inadequate health care resources, and also pointed to potential solutions to these problems, such as the use of videos in public places to increase awareness of cognitive impairment among the elderly. Subsequent symposia in Brazil and Argentina provided forums for a deeper examination of these issues, as well the communication of new directions in public policy in these countries.

During the development of all AAIC SS, one aim has been to select locations that permit greater involvement of regions comprising LMICs. The second AAIC SS meeting, for example, which took place in Bulgaria, in collaboration with researchers at the University of Varna and the University of Pittsburgh, brought this conversation closer to Eastern Europe, provided a forum for showcasing dementia research in neighboring countries, and engaged the expertise of leading researchers in that region of the world. In 2018, an AAIC SS meeting held in Bengaluru, India provided a forum for exploring emerging dementia research in South Asia and similarly engaged leading dementia researchers throughout that region, including in LMICs.

All meetings sponsored by the Alzheimer's Association, including AAIC and all satellite symposia, aim to encourage collaboration among professionals from all countries, including LMICs. In 2008, the Alzheimer's Association established a new means to support the dementia research community by creating the International Society to Advance Alzheimer's Research and Treatment (ISTAART) (24). This esteemed professional society for scientists, physicians, and other professionals interested in dementia science is the first international collegial organization to support and encourage the interests of all areas of AD and dementia investigation. In April 2019, ISTAART launched a new tiered dues structure for individuals from low, lower-middle and upper-middle economies (based on the World Bank's annual classification system). Since April 2019, ISTAART membership from low to upper middle income countries has nearly doubled, expanding access to ISTAART benefits spanning the Alzheimer's and Dementia journal family, Alzheimer's Association meetings, and Professional Interest Areas (PIAs) (24).

## Future Directions

In line with the UN's Sustainable Development Goal number three to ensure healthy lives and promote well-being for all at all ages, the global dementia research enterprise continues to strive for effective prevention and treatment throughout the world. A critical goal will be to sustain recent efforts to ensure inclusion of LMICs—where two-thirds of people with dementia reside—in large-scale, multinational research studies. Another important goal will be to identify modifiable risk factors in LMICs and determine practical, cost-effective ways of addressing them (3). Opportunities to seek funding and convene the dementia research community, with an emphasis on these areas, will continue to serve as a priority for the Association. Toward these ends, the Alzheimer's Association is committed to continuing its core mission of fostering and expanding partnerships, ensuring the continuation of LMIC-focused projects, identifying gaps in LMIC research and resources, and enhancing collaboration and communication among all researchers in LMIC regions. Such strategies are not independent, and it will be the sum of these approaches that will ultimately impact upon this goal.

## Data Availability Statement

The original contributions generated in the study are included in the article/supplementary material, further inquiries can be directed to the corresponding author.

## Author Contributions

HS, LC, and CS provided outline and specific details for sections of the manuscript. SW supported initial draft. MC oversight of the full manuscript and strategy development. All authors contributed to the article and approved the submitted version.

## Conflict of Interest

The authors declare that the research was conducted in the absence of any commercial or financial relationships that could be construed as a potential conflict of interest.
